# High-Altitude Drives the Convergent Evolution of Alpha Diversity and Indicator Microbiota in the Gut Microbiomes of Ungulates

**DOI:** 10.3389/fmicb.2022.953234

**Published:** 2022-07-07

**Authors:** Xibao Wang, Xiaoyang Wu, Yongquan Shang, Ying Gao, Ying Li, Qinguo Wei, Yuehuan Dong, Xuesong Mei, Shengyang Zhou, Guolei Sun, Lixian Liu, Bi Lige, Zhihao Zhang, Honghai Zhang

**Affiliations:** ^1^College of Life Sciences, Qufu Normal University, Qufu, China; ^2^Wild World Jinan, Jinan, China; ^3^Shijiazhuang Zoo, Shijiazhuang, China; ^4^Forestry and Grassland Station, Golmud, China

**Keywords:** gut microbiome, ungulates, phylogeny, high-altitude, convergent evolution

## Abstract

Convergent evolution is an important sector of evolutionary biology. High-altitude environments are one of the extreme environments for animals, especially in the Qinghai Tibet Plateau, driving the inquiry of whether, under broader phylogeny, high-altitude factors drive the convergent evolution of Artiodactyla and Perissodactyla gut microbiomes. Therefore, we profiled the gut microbiome of Artiodactyla and Perissodactyla at high and low altitudes using 16S rRNA gene sequencing. According to cluster analyses, the gut microbiome compositions of high-altitude Artiodactyla and Perissodactyla were not grouped together and were far from those of low-altitude Artiodactyla and Perissodactyla. The Wilcoxon’s test in high-altitude ungulates showed significantly higher Sobs and Shannon indices than in low-altitude ungulates. At the phylum level, Firmicutes and Patescibacteria were significantly enriched in the gut microbiomes of high-altitude ungulates, which also displayed a higher Firmicutes/Bacteroidetes value than low-altitude ungulates. At the family level, Ruminococcaceae, Christensenellaceae, and Saccharimonadaceae were significantly enriched in the gut microbiomes of high-altitude ungulates. Our results also indicated that the OH and FH groups shared two significantly enriched genera, *Christensenellaceae_R_7_group* and *Candidatus_Saccharimonas*. These findings indicated that a high altitude cannot surpass the order level to drive the convergent evolution of ungulate gut microbiome composition but can drive the convergent evolution of alpha diversity and indicator microbiota in the gut microbiome of ungulates. Overall, this study provides a novel perspective for understanding the adaptation of ungulates to high-altitude environments.

## Introduction

The gut microbiome can provide energy, in the form of short-chain fatty acids, to its host and maintain the gut homeostatic balance (Gonçalves et al., 2018; Markowiak-Kopeć and Śliżewska, 2020). Many factors affect gut microbiome composition and function, including diet (Huang et al., 2022), phylogeny (Wang et al., 2019; Gregor et al., 2022), and environment (Gacesa et al., 2022; Wang et al., 2022). When a factor surpasses the other influencing factors, the gut microbiome converges to adapt toward this dominant factor. Giant panda (*Ailuropoda melanoleuca*) and red panda (*Ailurus fulgens*) are typical models for convergent evolution studies. Huang et al. (2021) found that their bamboo diet, rather than host phylogeny, is the dominant driver of gut microbiome convergence within these species. Surprisingly, giant pandas and bamboo-eating insects share strikingly similar gut microbiota (Yao et al., 2021). Owing to their diets, whales have similar gut microbiome compositions to that of carnivores (Wu et al., 2022). Similarly, the gut microbiomes of many myrmecophagous species, such as anteaters, aardvarks, and aardwolves, are clustered in the same group, although they belong to different orders (Delsuc et al., 2014).

In addition to animals with specialized diets, species with closer phylogenetic relationships or similar behavior have similar gut microbiome compositions (Gregor et al., 2022). Amato et al. (2019) found that host phylogeny outweighs the dietary niche in structuring primate gut microbiomes. The phylogeny of water bird species and their gut microbiome hierarchical tree evinced phylosymbiosis (Laviad-Shitrit et al., 2019). Flight behavior drives the convergent evolution of the gut microbiome in bats and birds, where Proteobacteria are the dominant bacterial species (Song et al., 2020).

Many scholars have focused on the environmental impacts to the gastrointestinal microbiome of different species, especially in high-altitude environments. For example, based on a principal component analysis plot of high-altitude rumen, such as yak and Tibetan sheep, microbiome compositions are clustered, with significantly enriched VFA-yielding pathways (Zhang et al., 2016). The Firmicutes/Bacteroidetes ratio in high-altitude blue sheep and European mouflon is higher than in low-altitude blue sheep and European mouflon, and principal component analysis and non-metric multidimensional scaling (NMDS) plots have shown that the gut microbiomes of blue sheep and European mouflon at high-altitude are more similar than those of the same species at low altitude (Sun et al., 2019). Ma et al. (2019) found that Tibetan antelopes (*Pantholops hodgsonii*), Tibetan sheep (*Ovis aries*), and Tibetan wild ass (*Equus kiang*) had similar gut microbiome compositions, and Tibetan antelopes and sheep were annotated to carbohydrate and energy metabolism. In these studies, significantly enriched microbiota and metabolic pathways were related to animal energy acquisition. High altitudes involve extreme environmental conditions, such as low oxygen (Wang et al., 2021), thus, driving the convergent evolution of gastrointestinal microbiome compositions and functions, which can produce more energy to maintain life activities in harsh plateau environments.

Ungulates are widely distributed, easy to domesticate and breed, and can adapt to a variety of environments (Mas-Coma et al., 2009). Some studies have shown that the feeding niche overlap of high-altitude (Qinghai-Tibet Plateau) ungulates is low in such high-altitude areas (Harris and Miller, 1995; Shrestha et al., 2005; Cao et al., 2009; Shi et al., 2016). There are abundant wild ungulates on the Tibetan Plateau, including Artiodactyla, such as yak (*Bos grunniens*), Tibetan antelopes (*P. hodgsonii*), and blue sheep (*Pseudois nayaur*), and Perissodactyla, such as Tibetan wild ass (*E. kiang*). Ungulates represent an excellent model for studying the convergent evolution of high-altitude gut microbiomes. Many scholars have conducted in-depth research on the convergent evolution of ungulates in different altitude environments based on microbiomics; however, in these studies, the species phylogeny is closely related at the family or genus level. Further, several of these studies have significant limitations; for example, Ma et al. (2019) did not compare the gut microbiome of *E. kiang* at different altitudes and performed cluster analyses between groups. Under broader phylogeny, high altitudes drive the convergent evolution of ungulate gut microbiomes; however, this issue remains unclear. Based on these previous studies, we hypothesized that high altitudes drive the convergent evolution of ungulate gut microbiome diversity patterns, core microbiota, composition, and indicator species at each classification level. Therefore, we used the 16S rRNA gene to compare the gut microbiomes of high- and low-altitude ungulates. We aim to reveal the how influential altitude and phylogeny are to ungulate microbiome compositions at different altitudes.

## Materials and Methods

### Sample Collection

Samples of wild and captive ungulates were collected from Golmud (Qinghai Province, China) and Jinan (Shandong Province, China; Jinan wildlife and Jinan zoos), respectively. We collected samples from different wild ungulates and observed distinct individuals, immediately collecting excrement-core samples. Three observation points (point 1, *B. grunniens* and *E. kiang*; point 2, *P. nayaur*; and point 3, *P. hodgsonii*) along a straight-line distance of more than 100 km were considered. *B. grunniens* mainly feed on sedges in summer and graminoids in winter; *E. kiang* primarily consumes forbs in summer and browse in winter, while *P. hodgsonii* and *P. nayaur* primarily feed on graminoids and sedges in summer, respectively (Harris and Miller, 1995; Shi et al., 2016). Shi et al. (2016) observed that dietary overlaps were generally low among *P. hodgsonii*, *B. grunniens*, and *E. kiang* based on their Pianka’s indices. Captive ungulates ranged from 3 to 8 years old, were healthy, and had not received any medications within 3 months of sampling. The *B. grunniens*, *P. nayaur*, and *E. kiang* from the two zoos were fed approximately the same diets, consisting of cotton grass, alfalfa, carrots, and pellet feed. Information of all samples is detailed in Supplementary Tables S1, S2. All wild and captive samples were placed in sterile tubes and stored at −20°C during transit to Qufu Normal University, China, where all samples were frozen at −80°C until sequencing. This methodology followed the ethical standards of the Qufu Normal University Animal Care and Use Committee (Permit Number: 2022-020).

### DNA Extraction and High-Throughput Sequencing

Bacterial DNA was extracted from the samples with HiPure Stool DNA Mini Kits (Magen, Guangzhou, China) following the manufacturer’s protocols. We used a Qubit 2.0 fluorometer (Thermo Fisher Scientific, Waltham, MA, United States of America) to measure the DNA to ensure concentrations were higher than 20 ng/μl. The V3–V4 hypervariable regions of bacterial 16S rRNA genes were amplified using PCR primers (forward primer: CTACGGGNGGCWGCAG; reverse primer: GACTACHVGGGTATCTAATCC; Wu et al., 2016). We performed PCR amplification in a 50 μl reaction mixture containing 1.5 μl of 2.5 mM dNTPs, 10 μl of 5 × Q5 Reaction Buffer, 10 μl of 5 × Q5 High GC Enhancer, 0.2 μl of Q5 High-Fidelity DNA Polymerase, 1.5 μl of the forward and reverse primers, and 25.3 μl of DNA. The PCR conditions were as follows: pre-denaturation at 95°C for 5 min, with 30 cycles of 1 min at 95°C for denaturation, 30 cycles of 1 min at 60°C for annealing, 30 cycles of 1 min at 72°C for elongation, and a final elongation at 72°C for 5 min. A TruSeq^®^ DNA PCR-Free Sample Preparation Kit (Illumina, San Diego, CA, United States of America) was used to generate the DNA libraries, and the DNA library quality was quantified using a Qubit 2.0 Fluorometer (Thermo Fisher Scientific, Waltham, MA, United States of America) and FEMTO Pulse system (Agilent Technologies, Santa Clara, CA, United States of America). The DNA library was sequenced on an Illumina HiSeq platform (PE 250, United States of America) with a 250 bp.

### Sequence Processing and Statistical Analyses

The pair-ended reads were combined with raw tags using FLASH (v. 1.2.7; Magoč and Salzberg, 2011), and chimeric sequences were eliminated from them using the UCHIME algorithm (Edgar et al., 2011) to obtain effective tags. Based on a 16S rRNA sequence similarity at or greater than 97%, we performed UPARSE (v. 9.2.64; Edgar, 2013) to cluster the effective tags into operational taxonomic units (OTUs), and used the Mothur (Schloss et al., 2009) and SILVA databases (v 138.1; Quast et al., 2012) to annotate species with a threshold of 0.8–1. Alpha diversity [that is, the observed number of OTUs (Sobs), Chao1, Simpson, Shannon, and Good’s coverages] and beta diversity (that is, the Bray-Curtis distance) were calculated using the QIIME package (v. 1.9.1; Caporaso et al., 2010). Rarefaction curve and species accumulation boxplot analyses were performed with the Tutools platform (http://www.cloudtutu.com), a free online data analysis website. Principal coordinate analysis (PCoA), non-metric multidimensional scaling (NMDS), and the unweighted pair group method with arithmetic mean (UPGMA) were plotted using the ggplot2 package in R software (v 2.15.3). We used the Vega package in R software (v. 2.15.3) to perform Wilcoxon’s test (*p* < 0.05) and analysis of similarities, and used LDA Effect Size software (LDA score > 4, *p* < 0.05; White et al., 2009) to detect bacteria with significant differences between the groups.

## Results

### Overview of the Sequencing Data

**Figure 1:**
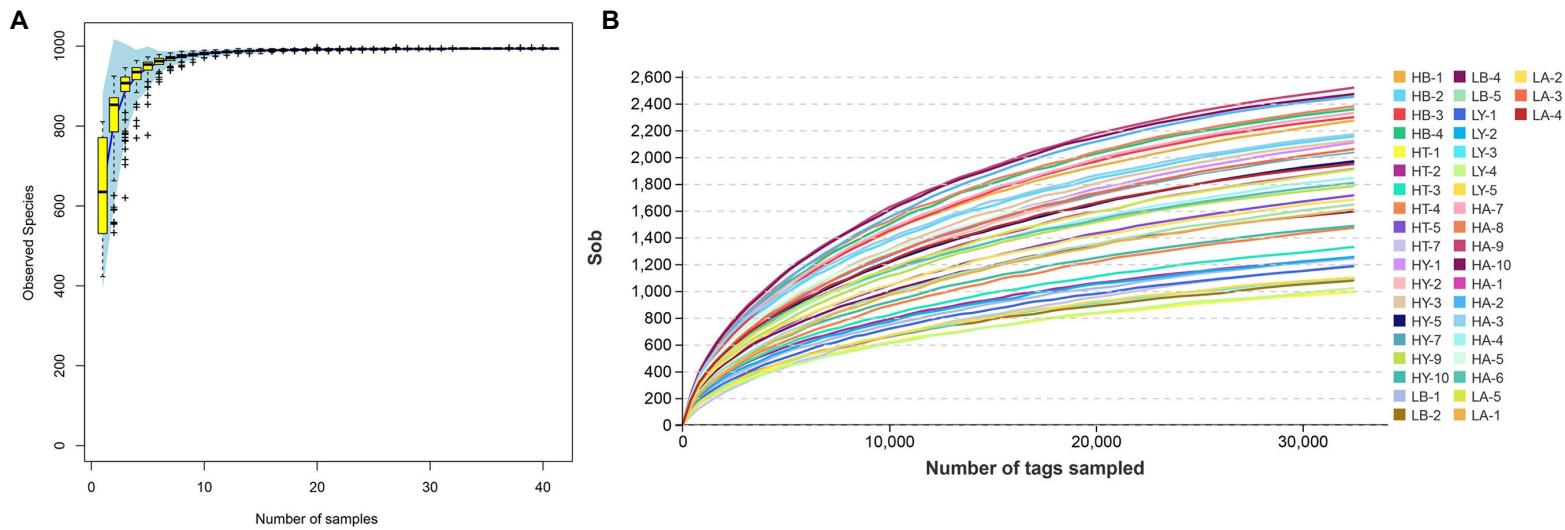
Species accumulation boxplot **(A)** and rarefaction curves **(B)**. In the species accumulation boxplot, the abscissa is the sample size, and the ordinate is the observed species. In the rarefaction curves, the abscissa is the number of sequencing samples randomly chosen from the sample, and the ordinate is the number of OTUs (Sobs).

We obtained 4,374,777 effective tags from 41 samples with an average of 106,702 effective tags per sample. With a 97% similarity criterion threshold, sample HA-9 showed the highest number of OTUs among all samples (2,560 OTUs), whereas sample HT-1 showed the lowest number of OTUs (1,161 OTUs; Supplementary Table S3, Sobs). Other alpha diversity indices are listed in Supplementary Table S3 (including Chao1, Simpson, Shannon, and Good’s coverages). The species accumulation boxplot and 41 rarefaction curves approached a plateau, suggesting that the number of samples and sequencing depth were sufficient for experimental analyses (Figure 1). Furthermore, the Good’s coverage index of each sample was higher than 95%, which also showed that the bacterial communities in the samples effectively represented those in the ungulate gut.

### Cluster Analyses

**Figure 2:**
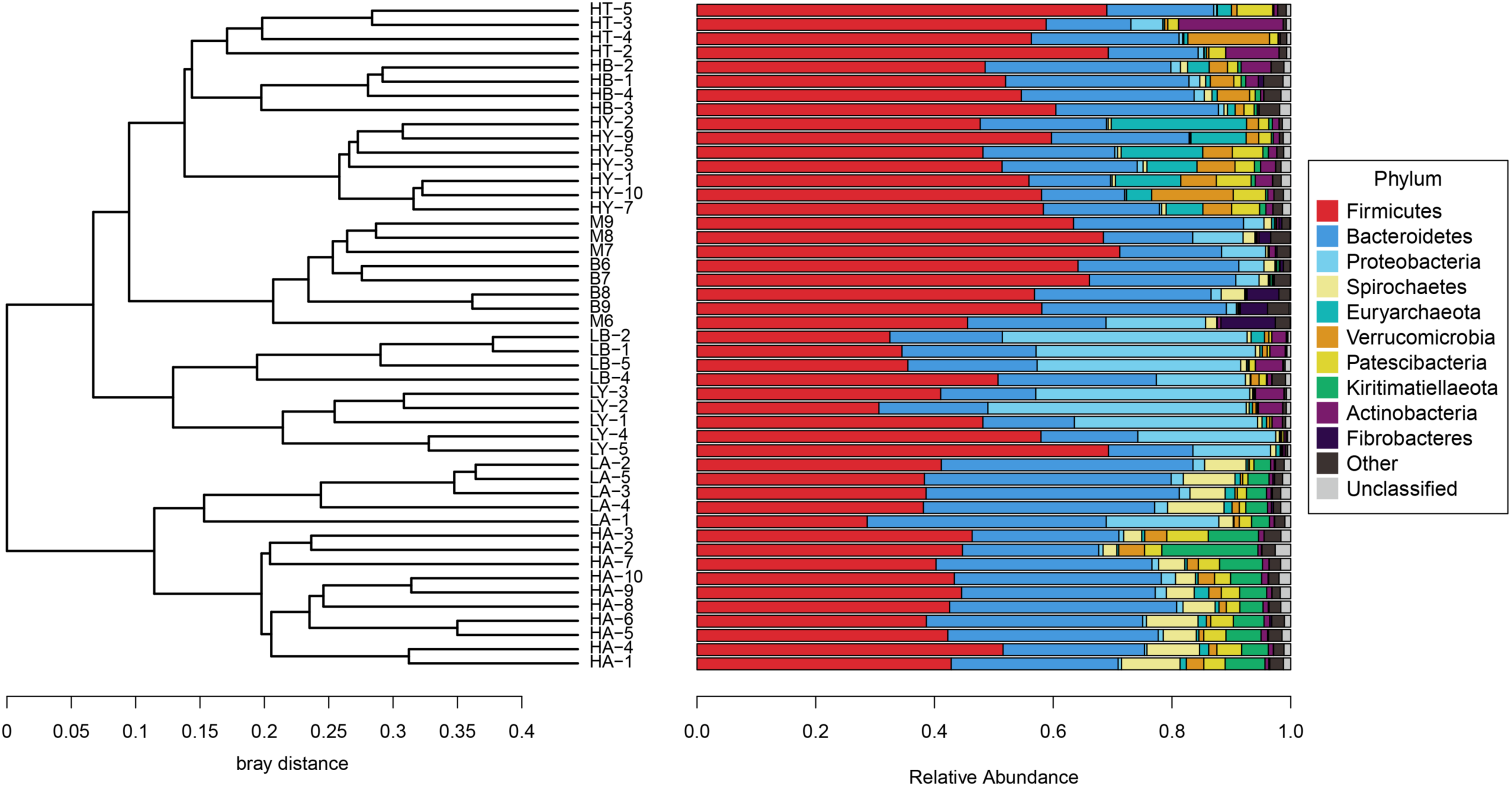
Cluster analysis with the Bray–Curtis distance. The unweighted pair group method with arithmetic mean (UPGMA) tree is generate based on the top 10 phyla.

**Figure 3:**
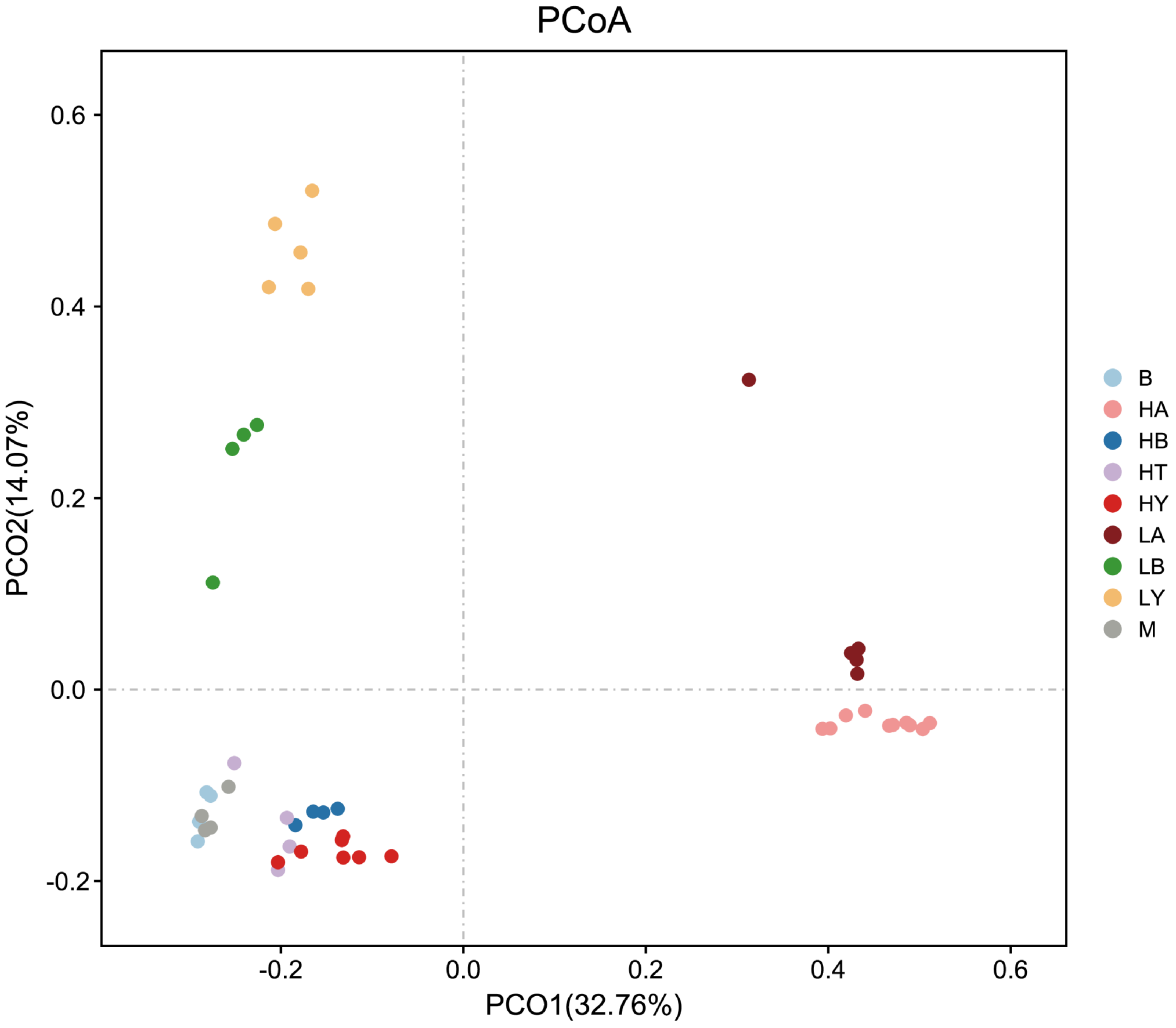
Principal coordinates analysis (PCoA) of gut microbiome composition. Each symbol represents the gut microbiome of a group. HA, high-altitude Tibetan wild ass; HB, high-altitude blue sheep; HT, high-altitude Tibetan antelope; HY, high-altitude yak; LA, low-altitude Tibetan wild ass; LB, low-altitude blue sheep; LY, low-altitude yak. B, blue sheep from Xining wildlife zoo, M, European Mouflon from Xining wildlife zoo.

**Figure 4:**
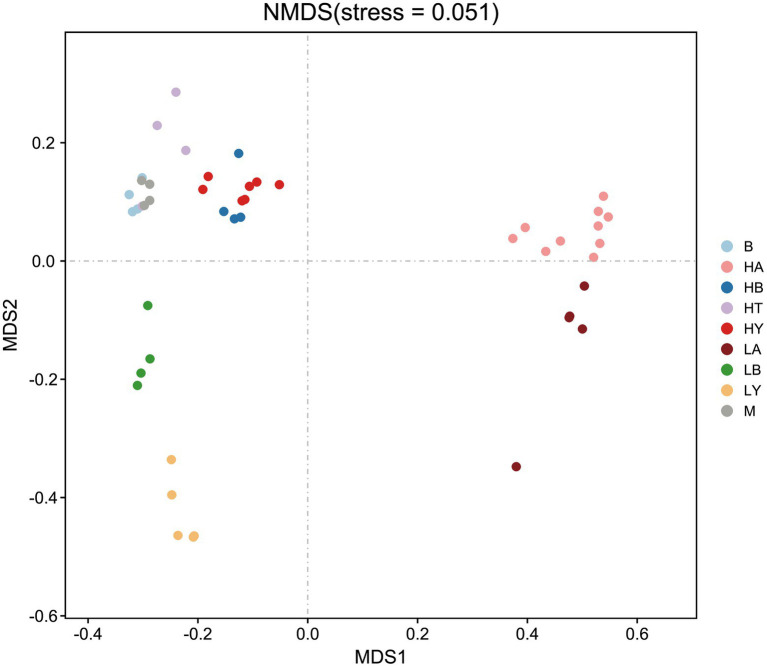
Non-metric multidimensional scaling (NMDS) of gut microbiome composition. Each symbol represents the gut microbiome of a group. HA, high-altitude Tibetan wild ass; HB, high-altitude blue sheep; HT, high-altitude Tibetan antelope; HY, high-altitude yak; LA, low-altitude Tibetan wild ass; LB, low-altitude blue sheep; LY low-altitude yak. B, blue sheep from Xining wildlife zoo, M, European Mouflon from Xining wildlife zoo.

Since food composition differed between wild and captive ungulates, we added the published data of blue sheep (Group B) and European Mouflon (Group M); (samples were collected from the Xining wildlife zoo) into the cluster analyses to eliminate dietary effects. The ungulates of the Xining wildlife zoo were fed approximately the same diets as those of the Jinan wild zoo and Jinan zoo [Sun et al., 2019; Supplementary Table S4, with previously published sample information (Sun et al., 2019)]. Based on the relative abundance at the phylum level and the Bray–Curtis distance, we selected the top 10 phyla to generate a UPGMA tree (Figure 2). Artiodactyla species (groups HT, HB, HY, M, B, LB, and LY) were grouped together, and those from Perissodactyla (groups HA and LA) were clustered into another clade. The Artiodactyla and Perissodactyla clades diverged at high and low altitudes. PCoA (Figure 3) and NMDS analysis (stress = 0.051, Figure 4) also showed that the gut microbiome compositions of Artiodactyla differed from those of Perissodactyla, and the gut microbiome compositions of high-altitude Artiodactyla (groups HT, HB, HY, M, and B) and Perissodactyla (group HA) differed greatly from those of low-altitude Artiodactyla (groups LB and LY) and Perissodactyla (group LA), respectively. These results showed that high altitudes could not surpass the order level to drive the convergent evolution of ungulate gut microbiome composition. Next, we eliminated dietary composition effects between wild and captive ungulates. Thus, we observed that the similarity of the gut microbiome composition corresponding to the HT, HB, HY, M, and B groups was higher than that corresponding to the LB, LY, and LA groups. This implied that the main influencing factors were altitude and phylogeny, and not diet. Therefore, based on these two influencing factors, we classified high-altitude Artiodactyla (groups HT, HB, and HY), high-altitude Perissodactyla (group HA), low-altitude Artiodactyla (groups LB and LY), and low-altitude Perissodactyla (group LA) as groups the FH, OH, FL, and OL groups, respectively.

The analysis of similarities (ANOSIM) demonstrated that the gut microbiome compositions of the OH and OL groups (*R* = 0.9695, *p* = 0.001) and the FH and FL groups (*R* = 0.7028, *p* = 0.001) showed significantly different unweighted uniFrac distances. This observation indicated that the division of these groups was reasonable.

### Gut Microbiome Composition

Overall, 25 phyla, 49 classes, 99 orders, 170 families, 366 genera, and 136 species were detected in the bacterial microbiome communities of the Perissodactyla and Artiodactyla samples.

**Figure 5:**
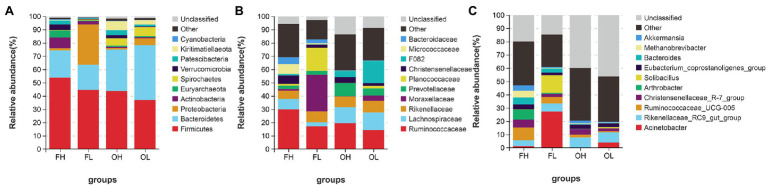
Gut microbiome composition of ungulates at the phylum **(A)**, family **(B)**, and genus **(C)** levels. FH, high-altitude Artiodactyla (including groups HB, HT, and HY); FL, low-altitude Artiodactyla (including groups LB and LY); OH, high-altitude Perissodactyla (group HA); OL, low-altitude Perissodactyla (group LA).

At the phylum level (top five abundance phyla), Firmicutes (F) and Bacteroidetes (B) dominated in groups such as FH (F, 53.77%; B, 20.57%), FL (F, 45.48%; B, 18.92%), OH (F, 43.71%; B, 31.35%), and OL (F, 38.98%; B, 41.5%). Other major phyla were Actinobacteria (7.99%), Euryarchaeota (5.27%), and Verrucomicrobia (4.13%) in the FH group; Proteobacteria (30.46%), Actinobacteria (2.41%), and Spirochaetes (0.72%) in the FL group; Kiritimatiellaeota (6.79%), Spirochaetes (5.62%), and Patescibacteria (3.68%) in the OH group; and Spirochaetes (6.71%), Proteobacteria (5.39%), and Kiritimatiellaeota (3.28%) in the OL group (Figure 5A).

At the family level, the top 10 families accounted for more than 59.26% of the four groups (FH: 69.19%; FL: 82.74%; OH: 59.26%; and OL: 66.72%). Among these 10 families, Ruminococcaceae was the most abundant in groups FH (29.98%) and OH (19.38%). Moraxellaceae (27.49%) was the most abundant in the FL group, and F082 (16.81%) was the most abundant in the OL group (Figure 5B). Additionally, at the genus level, *Ruminococcaceae_UCG-005* (9.44%) was the most abundant genus in the FH group, *Acinetobacter* (27.39%) showed predominance in the FL group, while *Rikenellaceae_RC9_gut_group* showed predominance in the OH (7.63%) and OL (7.70%) groups (Figure 5C).

### Analysis of Discrepancies for Between Groups

**Figure 6:**
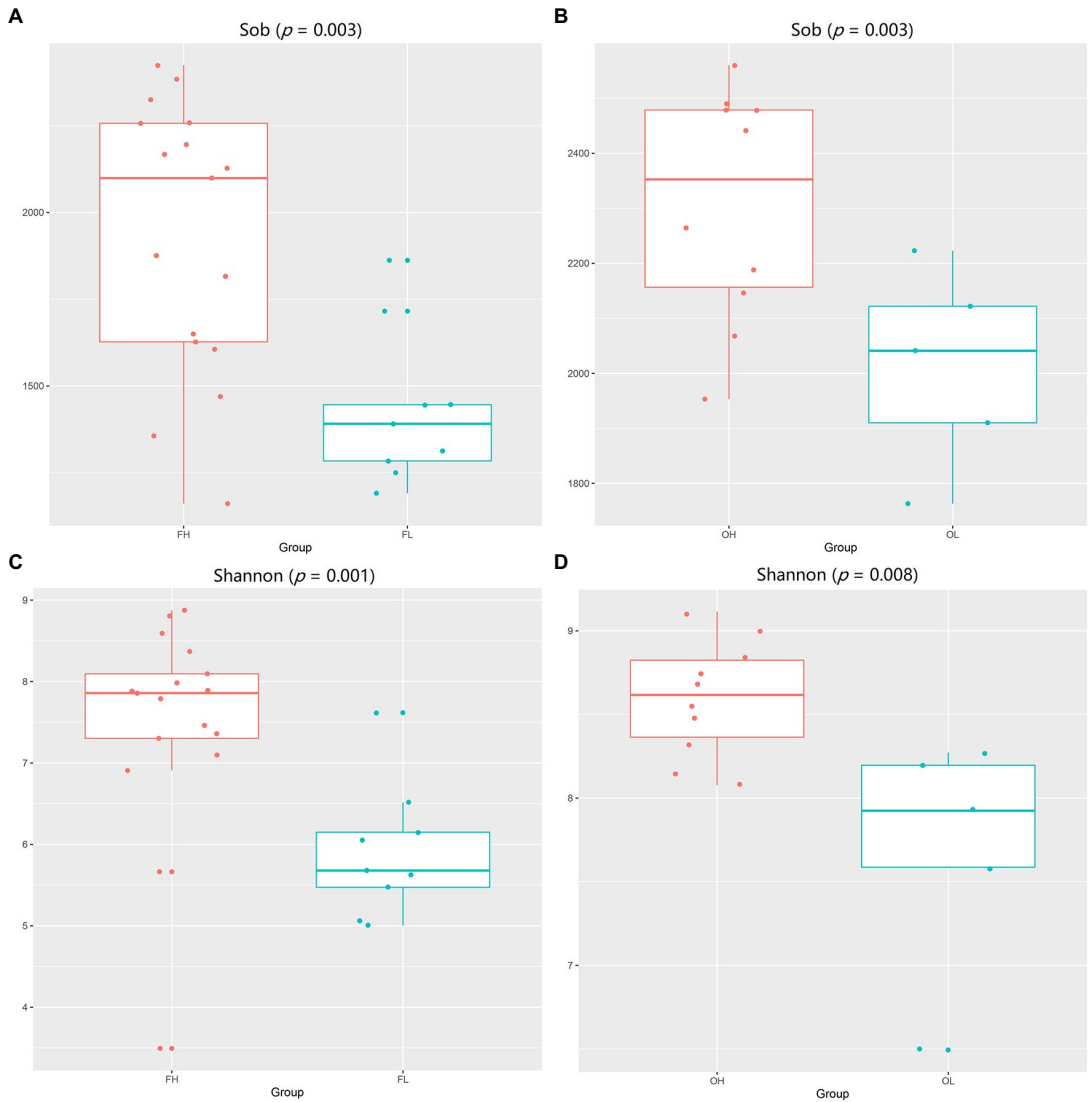
Wilcoxon’s test of alpha-diversity (Sobs and Shannon index) of gut microbiome between groups. **(A)** Sob index of group FH vs. group FL; **(B)** Sob index of group OH vs. group OL; **(C)** Shannon index of group FH vs. group FL; **(D)** Shannon index of group OH vs. group OL. FH, high-altitude Artiodactyla (including groups HB, HT, and HY); FL, low-altitude Artiodactyla (including groups LB and LY); OH, high-altitude Perissodactyla (group HA); OL, low-altitude Perissodactyla (group LA).

Wilcoxon’s test of Sobs (FH group vs. FL group, *p* = 0.003; OH group vs. OL group, *p* = 0.03) and Shannon (FH group vs. FL group, *p* = 0.001; OH group vs. OL group, *p* = 0.008) indices are shown in the boxplot (Figure 6). The gut microbiome diversity and richness of groups FH and OH were significantly higher than those of the low-altitude groups (FL and OL).

**Figure 7:**
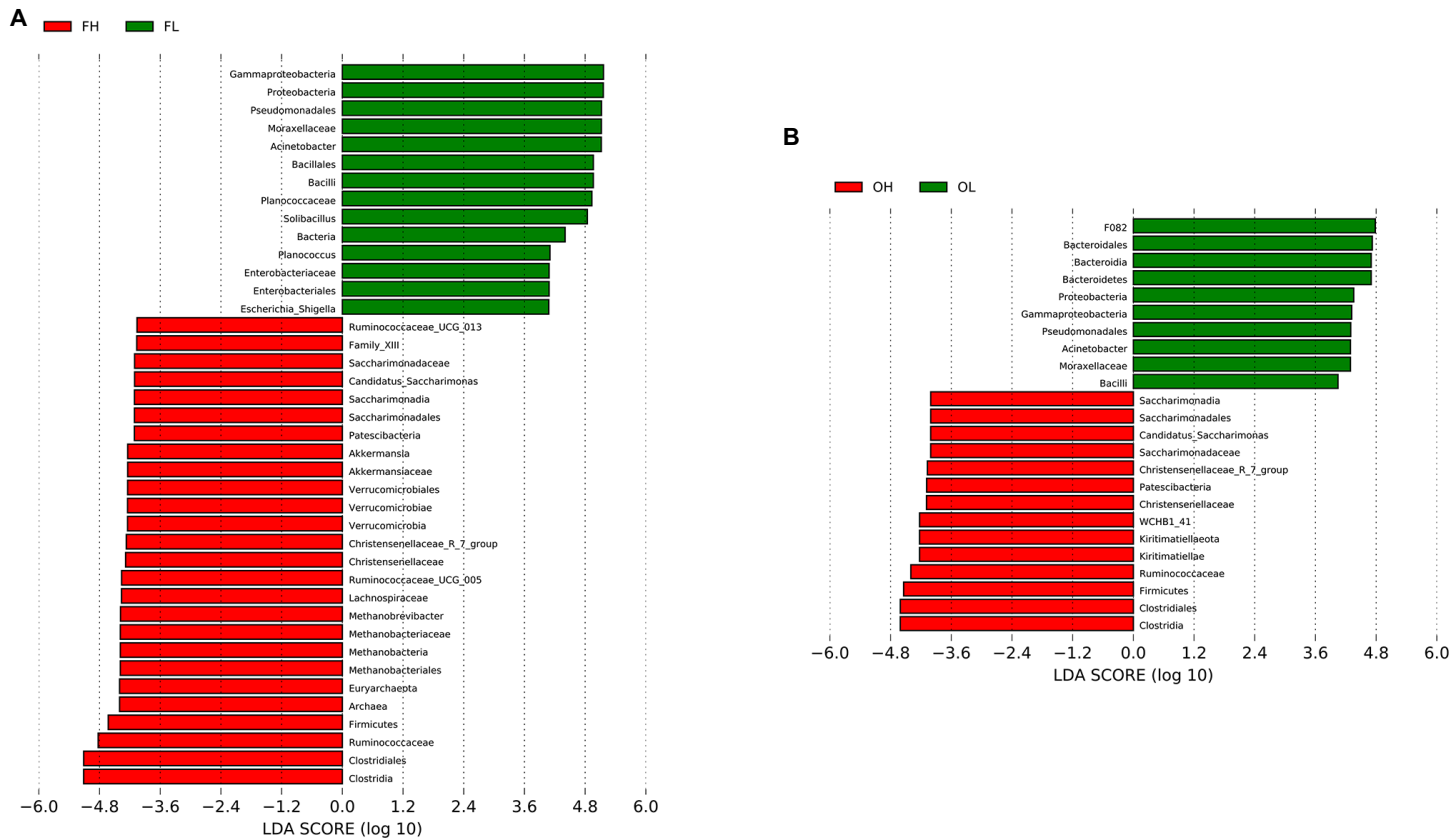
LEfSe (LDA Effect Size) analysis in ungulates between different altitudes. **(A)** Group FH (high-altitude Artiodactyla, including groups HB, HT, and HY) vs. group FL (low-altitude Artiodactyla, including groups LB and LY); **(B)** Group OH (high-altitude Perissodactyla, group HA) vs. group OL (low-altitude Perissodactyla, group LA).

We used LDA Effect Size analysis (LDA score > 4, *p* < 0.05) to identify the indicator microbiota (biomarker) between groups (groups FH and FL and groups OH and OL). At the phylum level, Firmicutes and Patescibacteria were significantly enriched in the FH and FL groups. Verrucomicrobia and Euryarchaeota were significantly enriched in the FH group. Meanwhile, Kiritimatiellaeota were more abundant in the OH group than in the OL group, Proteobacteria were significantly enriched in groups FL and OL, and Bacteroidetes were more abundant in the OL group than in the OH group (Figure 7).

At the family level, Ruminococcaceae, Christensenellaceae, and Saccharimonadaceae were significantly enriched in groups FH and OH; Akkermansiaceae, Lachnospiraceae, FamilyXIII, and Methanobacteriaceae were significantly enriched in the FH group. Moraxellaceae was significantly enriched in the FL and OL groups; Planococcaceae and Enterobacteriaceae were more abundant in the FL group than in the FH group; and F082 was more abundant in the OL group than in the OH group (Figure 7). At the genus level, *Christensenellaceae_R_7_group* and *Candidatus_Saccharimonas* were significantly enriched in the FH and OH groups. However, *Ruminococcaceae_UCG-013*, *Ruminococcaceae_UCG-005*, *Methanobrevibacter*, and *Akkermansia* were more abundant in the FH group than in the FL group. Our results also indicated that *Escherichia_Shiella*, *Acinetobacter*, *Solibacillus*, and *Planococcus* were more abundant in the FL group than in the FH group, and *Acinetobacter* was more abundant in the OL group than in the OH group (Figure 7).

Wilcoxon’s test was used to analyze the Firmicutes/Bacteroidetes (F/B) values between the groups. Supplementary Figure S1 shows that the F/B values of the high-altitude groups (FH and OH) were higher than those of the low-altitude groups (FL and OL; group FH vs. group FL, *p* > 0.05) and that of the OH group was significantly higher than that of the OL group (*p* < 0.01).

## Discussion

The gut microbiome is important for the ability of the host to adapt to various factors, including temperature, niche, and diet (Bo et al., 2019; Greene et al., 2020), which can provide energy and nutrients and maintain gut homeostasis in the host (Cani and Delzenne, 2007; Chung and Kasper, 2010; Wu et al., 2017). Many scholars have studied the adaptation mechanism of animals to high altitudes through the gut microbiome (Mazel, 2019; Xu et al., 2020), especially ungulates (Zhang et al., 2016, 2020; Sun et al., 2019). However, phylogeny has had little effect on their gut microbiome compositions in previous studies, because the phylogeny of ungulates was similar at the family, subfamily, and genus levels. Therefore, under broader phylogeny, this research explored the factors driving the convergent evolution of the gut microbiome of ungulates at different altitudes.

Using 16S rRNA high-throughput sequencing, we described the gut microbiome compositions of ungulates at different altitudes. At the phylum level, Firmicutes and Bacteroidetes were the dominant phyla in all samples (Figure 5). These results were consistent with other gut microbiomes of ungulates, such as the *E. kiang* (Gao et al., 2020), *B. grunniens* (Guo et al., 2021), *P. hodgsonii* (Bai et al., 2018), and *P. nayaur* (Sun et al., 2019).

According to the UPGMA tree, we found that groups HA, HB, HT, and HY were not clustered in the clade, while Artiodactyla (groups HB, HT, HY, LY, M, B, and LB) were clustered together. Ungulates with close phylogenetic relationships had similar gut microbiome compositions. These results revealed that high altitudes could not surpass the order level to drive the convergent evolution of the relative abundance composition of the ungulate. Phylogeny is one of the main factors affecting the gut microbiome composition of species (Benson et al., 2010; Zhu et al., 2021); for example, a study of 59 neotropical bird species found that the phylogeny factor was most frequently significant and explained most of the variation (Hird et al., 2015). In different niches, non-primates with close phylogenetic relationships possess similar gut microbiome compositions (Amato et al., 2019). Furthermore, the gut microbiome composition of Artiodactyla and Perissodactyla differed at high or low altitudes, and groups HB, HT, HY, M, and B clustered together through PCoA and NMDS plots. This showed that high altitudes drove the convergent evolution of ungulate gut microbiome composition at the order level. Notwithstanding, altitude is the main factor that affects the relative microbiome composition of ungulates. This is because, even though the diets of corresponding to the Xining wildlife zoo groups (the M and B groups) and the FL group were similar in composition, they did not group together.

High-altitude groups (OH and FH) were significantly higher than the low-altitude groups (FL and OL) in the Wilcoxon’s test of Sobs and Shannon. Higher gut bacterial diversity and richness generally correlate with a healthy and stable host gut microbiome (Petersen and Round, 2014; Waite and Taylor, 2014). High altitudes drove the gut microbiome of Artiodactyla and Perissodactyla to produce the same pattern of alpha diversity.

In this study, the OH and FH groups harbored large proportions of Firmicutes, which can decompose fibers and cellulose to provide volatile fatty acids to the host (Allison et al., 1962; Dai et al., 2015). Additionally, the F/B values of the high-altitude groups showed an increasing trend compared with that of the low-altitude groups. These high F/B values indicate that the host has a large body size and fat (Magne et al., 2020; Wolf et al., 2021). Many researchers have found that the F/B value of high-altitude ungulate gastrointestinal tract microbiomes was significantly higher than that of low-altitude ungulates (Ma et al., 2019; Sun et al., 2019). A high F/B ratio is beneficial for high-altitude ungulate energy harvesting (Zhang et al., 2016; Lan et al., 2017). The relative abundances of Patescibacteria in the OH and FH groups were higher than those in the OL and FL groups. Nitrate reductase (EC 1.7.99.4) positively predicts nitrite reductase (NO-forming, EC 1.7.2.1) and nitric-oxide reductase (cytochrome c, EC 1.7.2.5) in Patescibacteria, which has a synergistic effect on denitrification (Yang et al., 2022). Denitrification bacteria can reduce methane gas production in cow rumen liquids (Megga et al., 2017), as methane is a rumen fermentation by-product from methanogenic archaea, which leads to energy loss (Morgavi et al., 2010). Patescibacteria might reduce the energy loss of ungulates in high-altitude environments.

The relative abundance of Verrucomicrobia and Euryarchaeota was significantly higher in the FH group than in the OH group. Verrucomicrobia exists in the gut mucosa of healthy individuals and has anti-inflammatory effects (Fujio-Vejar et al., 2017) while maintaining glucose homeostasis in the host microbiome (Lindenberg et al., 2019). Euryarchaeota can promote carbohydrate fermentation to produce short-chain fatty acids, which play an important role in the gut ecosystem (Chaudhary et al., 2018). Kiritimatiellaeota is the major microbiota in horse (Edwards et al., 2020) and wild gelada (Baniel et al., 2021) hindguts and exists in the rumen of sheep (Wang et al., 2017) and cattle (Ribeiro et al., 2017). We speculate that Kiritimatiellaeota plays an important role in the digestion of herbivorous species, which requires further functional verification.

Ruminococcaceae, Christensenellaceae, and Saccharimonadaceae were characterized by a significant increase in the OH and FH groups. Ruminococcaceae and Christensenellaceae were the top 10 families in groups OH and FH. Ruminococcaceae is related to the decomposition of cellulose and starch (Andersen-Ranberg et al., 2018) and can produce acetic and formic acids (Ze et al., 2012). Christensenellaceae is an efficient sugar-fermenting family that can decompose glucose into acetic and butyric acids and can decompose cellulose (Morotomi et al., 2012). Meanwhile, Saccharimonadaceae is associated with immune response (Zhao et al., 2020). FamilyXIII is associated with the maintenance of gut health (Zhao et al., 2020), and Lachnospiraceae contained several cellulolytic/fibrolytic genera, and its abundance increased in the FH group, which is conducive to obtaining energy and maintaining homeostasis for the host in extreme environments (Biddle et al., 2013).

The OH and FH groups shared two significantly enriched genera, *Christensenellaceae_R_7_group* and *Candidatus_Saccharimonas*. The function of *Christensenellaceae_R_7_group* is the same as that of Christensenellaceae (Morotomi et al., 2012). Additionally, *Candidatus_Saccharimonas* species are positively correlated with propionic acid and butyric acid levels (Xia et al., 2022) and play an important role in maintaining gut functions (Xie et al., 2021). The relative abundance of the genus *Akkermansia* was greater in the FH group than in the other groups. Previous studies have shown that *Akkermansia* can consolidate the intestinal barrier and regulate immune functions (Zhao et al., 2022). Thus, the significantly enriched genera can help the high-altitude ungulates cope in extreme environments.

Based on the above results, the significantly enriched microbiota in high-altitude ungulates play an important role in disintegrating dietary fiber and cellulose, gaining energy, and maintaining gut homeostasis. High-altitude factors cannot surpass phylogeny to drive the convergent evolution of gut microbiome composition in ungulates, and high-altitude factors drive convergent evolution of alpha diversity and indicator microbiota in the gut microbiome of Artiodactyla and Perissodactyla at high altitudes. However, our understanding of the enriched microbiota and species level is still limited considering the limitations of 16S rRNA sequencing technology. Thus, in future studies, it would be necessary to employ metagenomics and metabolomics comprehensively to clarify the composition and function of enriched microbiota, especially at the species level, in ungulates at different altitudes.

## Data Availability Statement

The datasets presented in this study can be found in online repositories. The names of the repository/repositories and accession number(s) can be found at: https://www.ncbi.nlm.nih.gov/, PRJNA837737.

## Ethics Statement

The animal study was reviewed and approved by Qufu Normal University Animal Care and Use Committee (Permit Number: 2022-020).

## Author Contributions

XWa: conceptualization, methodology, data curation, software, and writing-original draft preparation. XWu: methodology and writing—review and editing. YS: writing—review and editing. YG and YL: sample provision. QW: conceptualization and resources. XM: visualization. YD, SZ, and ZZ: conceptualization. GS: data curation. LL and BL: investigation. HZ: conceptualization and writing—review and editing. All authors contributed to the article and approved the submitted version.

## Funding

This work was supported by the National Natural Science Foundation of China (32070405, 31872242, and 32001228).

## Conflict of Interest

The authors declare that the research was conducted in the absence of any commercial or financial relationships that could be construed as a potential conflict of interest.

## Publisher’s Note

All claims expressed in this article are solely those of the authors and do not necessarily represent those of their affiliated organizations, or those of the publisher, the editors and the reviewers. Any product that may be evaluated in this article, or claim that may be made by its manufacturer, is not guaranteed or endorsed by the publisher.

## Supplementary Material

The Supplementary Material for this article can be found online at: https://www.frontiersin.org/articles/10.3389/fmicb. 2022.953234/full#supplementary-material
