# Supplementary material for: High-Altitude Drives the Convergent Evolution of Alpha Diversity and Indicator Microbiota in the Gut Microbiomes of Ungulates
Source: Front Microbiol. 2022 Jul 7;13:953234. doi: 10.3389/fmicb.2022.953234 (PMC9301279; doi:10.3389/fmicb.2022.953234)
Supplement: Supplementary file 1 [file Table_1.DOCX]

TableS1 The samples information of Ungulata in high-altitude

| Species | Altitude | Longitude (E) | Latitude (N) | Sample name | Group |
| --- | --- | --- | --- | --- | --- |
| Blue sheep (*Pseudois nayaur*) | 3886 | 94.0388° | 35.91339° | HB-1 | HB |
|  |  |  |  | HB-2 |  |
|  |  |  |  | HB-3 |  |
|  |  |  |  | HB-4 |  |
| Tibetan Wild Ass (*Equus kiang*) | 3955 | 95.2525° | 35.77952° | HA-1 | HA |
|  |  |  |  | HA-2 |  |
|  |  |  |  | HA-3 |  |
|  |  |  |  | HA-4 |  |
|  |  |  |  | HA-5 |  |
|  |  |  |  | HA-6 |  |
|  |  |  |  | HA-7 |  |
|  |  |  |  | HA-8 |  |
|  |  |  |  | HA-9 |  |
|  |  |  |  | HA-10 |  |
| Yak (*Bos grunniens*) | 3955 | 95.2525° | 35.77952° | HY-1 | HY |
|  |  |  |  | HY-2 |  |
|  |  |  |  | HY-3 |  |
|  |  |  |  | HY-4 |  |
|  |  |  |  | HY-5 |  |
|  |  |  |  | HY-6 |  |
|  |  |  |  | HY-7 |  |
|  |  |  |  | HY-8 |  |
|  |  |  |  | HY-9 |  |
|  |  |  |  | HY-10 |  |
| Tibetan antelope (*Pantholops hodgsonii*) | 3773 | 91.90771° | 37.06834° | HT-1 | HT |
|  |  |  |  | HT-2 |  |
|  |  |  |  | HT-3 |  |
|  |  |  |  | HT-4 |  |
|  |  |  |  | HT-5 |  |
|  |  |  |  | HT-6 |  |
|  |  |  |  | HT-7 |  |

Table S2 The samples information of Ungulata in low-altitude

| Species | Altitude | Place | Sample name | Sex (M/F) | Group |
| --- | --- | --- | --- | --- | --- |
| Yak (*B. grunniens*) | 151 | Ji’nan Wild Zoo | LY-1 | M | LY |
|  |  |  | LY-2 | F |  |
|  |  |  | LY-3 | F |  |
|  |  |  | LY-4 | F |  |
|  |  |  | LY-5 | M |  |
| Blue Sheep (*P. nayaur*) | 30 | Ji’nan Wild Zoo | LB-1 | M | LB |
|  |  |  | LB-2 | M |  |
|  |  |  | LB-4 | F |  |
|  |  |  | LB-5 | F |  |
| [Tibetan Wild Ass](https://www.researchgate.net/publication/342762448_Comparison_of_the_Gut_Microbiota_in_the_Tibetan_Wild_Ass_Equus_kiang_Collected_from_High_and_Low_Altitude?_sg%5B0%5D=5tt2EQ4PqJcpVOmGzm9sruV5_h7VHPZ1UWyk5ztDPEiuhNOW5hRcwVuW5ZYa9f4QAthHX_IZf1pHQZNC__O0IgWB4qsI0CEezS_-7F-w.dClQAumoKoa6HEzFJG5CEJPk8jTfIGQ3-uMDqWs6z0AfuIqNIVsTv3egS-hwke5ns0K3aeUIvfXolt703_BQCg) (*E. kiang*) |  |  | LA-1 | M | LA |
|  |  |  | LA-2 | F |  |
|  |  |  | LA-3 | F |  |
|  |  |  | LA-4 | F |  |
|  |  |  | LA-5 | F |  |

Table S3 Alpha diversity of samples

| Samples | Sobs | Shannon | Simpson | Chao1 | Ace | Goods_coverage |
| --- | --- | --- | --- | --- | --- | --- |
| HT-1 | 1161 | 5.6640415 | 0.902870941 | 1247.542857 | 1344.95082 | 0.995839852 |
| HT-2 | 1356 | 7.3021119 | 0.980736169 | 1438.565789 | 1528.090706 | 0.99445034 |
| HT-3 | 1470 | 6.9102005 | 0.957780476 | 1640.625 | 1763.852656 | 0.992453885 |
| HT-4 | 1650 | 7.0983547 | 0.977250774 | 1832.877315 | 1969.646806 | 0.991622464 |
| HT-5 | 1876 | 7.8905656 | 0.986533371 | 2097.37 | 2266.374815 | 0.989264957 |
| HT-7 | 1606 | 3.4940733 | 0.617137271 | 1902.467422 | 2014.867315 | 0.993695541 |
| HY-1 | 2257 | 7.8824222 | 0.979963192 | 2475.407591 | 2617.290545 | 0.987714402 |
| HY-2 | 2196 | 7.3567744 | 0.949800103 | 2354.848175 | 2522.326424 | 0.988753763 |
| HY-3 | 2167 | 7.9803168 | 0.982298758 | 2391.409015 | 2553.171079 | 0.985251073 |
| HY-5 | 2128 | 7.8578522 | 0.975987582 | 2350.704918 | 2522.160122 | 0.988490246 |
| HY-7 | 2099 | 8.0946862 | 0.985932564 | 2328.725664 | 2516.048054 | 0.986016287 |
| HY-9 | 1816 | 7.7891717 | 0.9812189 | 1973.664804 | 2114.93302 | 0.988253407 |
| HY-10 | 1627 | 7.4608055 | 0.977577158 | 1764.927152 | 1908.693064 | 0.99215269 |
| HA-1 | 2146 | 8.0777114 | 0.988644418 | 2309.3157 | 2461.423027 | 0.991169177 |
| HA-2 | 2490 | 8.6724311 | 0.989715888 | 2590.384615 | 2730.955116 | 0.988539814 |
| HA-3 | 2264 | 8.7404259 | 0.992081925 | 2390.610015 | 2543.607061 | 0.989601078 |
| HA-4 | 2068 | 8.1579764 | 0.989877141 | 2245.381919 | 2399.490197 | 0.991008152 |
| HA-5 | 2188 | 8.4918485 | 0.991449148 | 2329.456 | 2499.919211 | 0.990082684 |
| HA-6 | 1953 | 8.32155 | 0.991476385 | 2126.084211 | 2259.694787 | 0.990833559 |
| HA-7 | 2479 | 8.5608679 | 0.988766001 | 2626.777778 | 2779.844889 | 0.989167874 |
| HA-8 | 2441 | 8.8528083 | 0.993152733 | 2651.320182 | 2827.091481 | 0.985422257 |
| HA-9 | 2560 | 9.1148692 | 0.99502248 | 2740.008097 | 2912.44824 | 0.985256794 |
| HA-10 | 2478 | 8.9982687 | 0.99424964 | 2648.693793 | 2820.263087 | 0.984876093 |
| HB-1 | 2424 | 8.8749848 | 0.99377055 | 2610.543269 | 2771.185267 | 0.988643044 |
| HB-2 | 2259 | 8.5929091 | 0.991327159 | 2459.069324 | 2622.867233 | 0.98779931 |
| HB-3 | 2325 | 8.3698095 | 0.984944449 | 2483.226365 | 2685.595627 | 0.98570801 |
| HB-4 | 2384 | 8.8089061 | 0.992456799 | 2579.900446 | 2760.564535 | 0.985004522 |
| LY-1 | 1391 | 5.4727699 | 0.896162528 | 1491.131926 | 1588.059756 | 0.995165359 |
| LY-2 | 1445 | 6.1495795 | 0.924304106 | 1538.757576 | 1644.671559 | 0.995023515 |
| LY-3 | 1250 | 5.6304351 | 0.911432146 | 1333.042017 | 1427.613964 | 0.995318765 |
| LY-4 | 1191 | 5.0050001 | 0.863033167 | 1289.480392 | 1360.071323 | 0.995690561 |
| LY-5 | 1284 | 5.0620151 | 0.830412953 | 1419.090361 | 1531.635364 | 0.99436016 |
| LB-1 | 1446 | 6.0526017 | 0.881975274 | 1572.160714 | 1680.864015 | 0.994159745 |
| LB-2 | 1313 | 5.6788305 | 0.854515071 | 1438.938596 | 1560.693581 | 0.995222154 |
| LB-4 | 1716 | 7.6166166 | 0.976779599 | 1807.475499 | 1945.207852 | 0.992710268 |
| LB-5 | 1862 | 6.5141954 | 0.899574538 | 1982.429319 | 2125.711879 | 0.992589346 |
| LA-1 | 1763 | 6.4937746 | 0.934834526 | 1918.46723 | 2032.527563 | 0.991598293 |
| LA-2 | 1910 | 7.5865231 | 0.971672294 | 2066.301181 | 2210.04697 | 0.992171098 |
| LA-3 | 2223 | 8.1961948 | 0.984650913 | 2403.516908 | 2571.185686 | 0.989068014 |
| LA-4 | 2041 | 8.2720898 | 0.990694034 | 2180.5625 | 2332.172061 | 0.989599836 |
| LA-5 | 2122 | 7.9244136 | 0.981464554 | 2277.016807 | 2433.173038 | 0.991093621 |


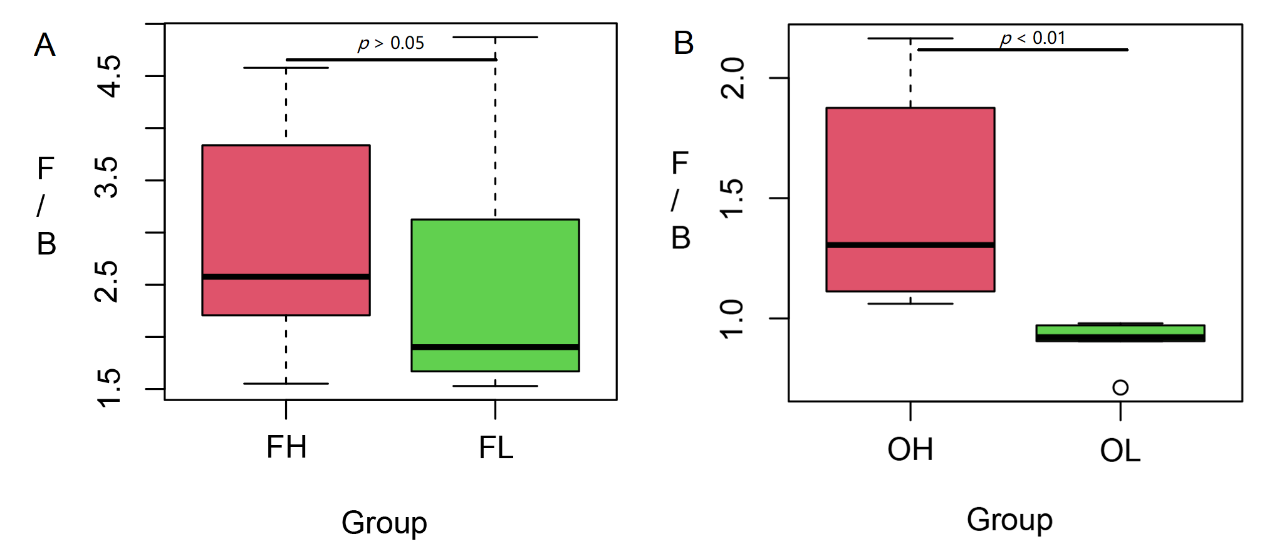
Table S4 The samples information of Xining wildlife zoo

| Species | Altitude | Sample name | Sex (M/F) | Group |
| --- | --- | --- | --- | --- |
| European mouflon (*Ovis musimon*) | 2348 | M6 | F | M |
|  |  | M7 | F | M |
|  |  | M8 | M | M |
|  |  | M9 | M | M |
| Blue sheep (*P. nayaur*) |  | B6 | M | B |
|  |  | B7 | M | B |
|  |  | B8 | F | B |
|  |  | B9 | F | B |

Fig. S1 Wilcoxon’s test of Firmicutes/Bacteroidetes (F/B) values between groups. FH, high-altitude Artiodactyla (including groups HB, HT, and HY); FL, low-altitude Artiodactyla (including groups LB and LY); OH, high-altitude Perissodactyla (group HA); OL, low-altitude Perissodactyla (group LA).
